# A whole canopy gas exchange system for the targeted manipulation of grapevine source-sink relations using sub-ambient CO_2_

**DOI:** 10.1186/s12870-019-2152-9

**Published:** 2019-12-03

**Authors:** Jason P. Smith, Everard J. Edwards, Amanda R. Walker, Julia C. Gouot, Celia Barril, Bruno P. Holzapfel

**Affiliations:** 1 National Wine and Grape Industry Centre, Wagga Wagga, New South Wales 2678 Australia; 2 Department of General and Organic Viticulture, Hochschule Geisenheim University, Von-Lade-Strasse 1, D-65366 Geisenheim, Germany; 3 Present Address: Current Address: Faculty of Science, Charles Sturt University, Leeds Parade, Orange, New South Wales 2800 Australia; 4 CSIRO Agriculture & Food, Locked Bag 2, Glen Osmond, South Australia 5064 Australia; 5 School of Agricultural and Wine Sciences, Faculty of Science, Charles Sturt University, Wagga Wagga, New South Wales 2678 Australia; 6 New South Wales Department of Primary Industries, Wagga Wagga, New South Wales 2678 Australia

**Keywords:** Grapevine, Source-sink relations, Grape berry, Sugar, CO_2_, Photosynthesis, Transpiration

## Abstract

**Background:**

Elucidating the effect of source-sink relations on berry composition is of interest for wine grape production as it represents a mechanistic link between yield, photosynthetic capacity and wine quality. However, the specific effects of carbohydrate supply on berry composition are difficult to study in isolation as leaf area or crop adjustments can also change fruit exposure, or lead to compensatory growth or photosynthetic responses. A new experimental system was therefore devised to slow berry sugar accumulation without changing canopy structure or yield. This consisted of six transparent 1.2 m^3^ chambers to enclose large pot-grown grapevines, and large soda-lime filled scrubbers that reduced carbon dioxide (CO_2_) concentration of day-time supply air by approximately 200 ppm below ambient.

**Results:**

In the first full scale test of the system, the chambers were installed on mature Shiraz grapevines for 14 days from the onset of berry sugar accumulation. Three chambers were run at sub-ambient CO_2_ for 10 days before returning to ambient. Canopy gas exchange, and juice hexose concentrations were determined. Net CO_2_ exchange was reduced from 65.2 to 30 g vine^− 1^ day^− 1^, or 54%, by the sub-ambient treatment. At the end of the 10 day period, total sugar concentration was reduced from 95 to 77 g L^− 1^ from an average starting value of 23 g L^− 1^, representing a 25% reduction. Scaling to a per vine basis, it was estimated that 223 g of berry sugars accumulated under ambient supply compared to 166 g under sub-ambient, an amount equivalent to 50 and 72% of total C assimilated.

**Conclusions:**

Through supply of sub-ambient CO_2_ using whole canopy gas exchange chambers system, an effective method was developed for reducing photosynthesis and slowing the rate of berry sugar accumulation without modifying yield or leaf area. While in this case developed for further investigations of grape and wine composition, the system has broader applications for the manipulation and of study of grapevine source-sink relations.

## Background

In the wine industry, where high yields are often perceived to be negatively associated with quality, there is a need to better understand how source-sink relations and the availability of carbohydrates during ripening influences berry composition and wine quality attributes. Firstly, because significant resources can be devoted to the management of canopy structure, fruit light exposure and yield [1–3], and knowledge of processes being targeted is important for predicting potential benefits for fruit composition and wine quality. Secondly, where the viticulture system, water availability, and climate allows it, higher yields provide an opportunity to increase production efficiency if quality can otherwise be maintained. A key challenge in understanding the yield-quality relationship is determining the extent to which berry metabolites are directly influenced by the availability of carbohydrates [4, 5], and separating these from responses to fruit exposure that may occur when canopy or crop adjustments are made [6–9].

Crop reductions in the form of shoot or bunch thinning have been demonstrated to improve aspects of berry composition and prevent delays in ripening in particularly high-yielding seasons [10, 11]. Conversely, as many wine producing regions face advanced ripening as the climate warms, the rate of sugar accumulation can be slowed by reducing the leaf area and photosynthetic output of the canopy [12–14]. However, in applying such treatments it can be difficult to ensure the fruit microclimate remains exactly the same across treatments, and compensatory responses may offset the intended effect on source-sink relations. Photosynthesis may be upregulated following leaf removal, while stored reserves can provide an alternative source of carbohydrates if photosynthetic supply is insufficient [15, 16]. If fruit is instead removed to increase sugar accumulation in remaining berries, this has been shown to have the desired effect in some studies [10], but minimal effect in other situations where yield or photosynthetic carbohydrate supply are apparently not limiting [17, 18].

An alternative method for the manipulation of whole plant carbon balance involves scaling up the system widely employed with single leaf gas exchange instruments, and modifying net canopy carbon assimilation by varying the amount of CO_2_ available for photosynthesis. Such an approach has been used for short term gas exchange measurements of whole tree canopies [19], with CO_2_ added to the air supply of two large chambers after scrubbing through columns of soda-lime. A similar capacity to vary CO_2_ supply above or below ambient for more extended periods with grapevines could provide a method of modifying photosynthetic carbohydrate supply relative to berry demand without the need to remove fruit or leaves. This would address the issue of fruit exposure changes, and combined with measurements of whole canopy gas exchange, the effects on net canopy photosynthesis in relation to effects on berry sugar accumulation could also be quantified.

The aim of this study was to build and validate a multi-chamber system with CO_2_ scrubbing capacity to enclose the canopies of mature fruiting grapevines that were grown outside in large pots. The chambers were built to complement field based studies into crop load and fruit composition, and to provide a method to examine the relationship between berry sugar accumulation and key primary and secondary metabolites in the absence of fruit exposure differences. Although CO_2_ re-injection was tested, and could be readily added to the system for routine use, the initial series of experiments undertaken were run at ambient and sub-ambient CO_2_ concentrations. In development of the CO_2_ scrubbing system a target reduction of 200 ppm, approximately half of ambient, was conceived to match the crop load variation that could be generated with commercially realistic crop or leaf area adjustments in the field. It also orientated the work towards the question of high yield effects on berry composition. In this paper, the design details of the system and its operation are described. To demonstrate the effectiveness of the approach for reducing photosynthesis and slowing ripening, gas exchange and berry sugar results are presented from the first experiment with the system conducted at onset of véraison.

## Results and discussion

### Chamber environmental conditions

**Fig. 1:**
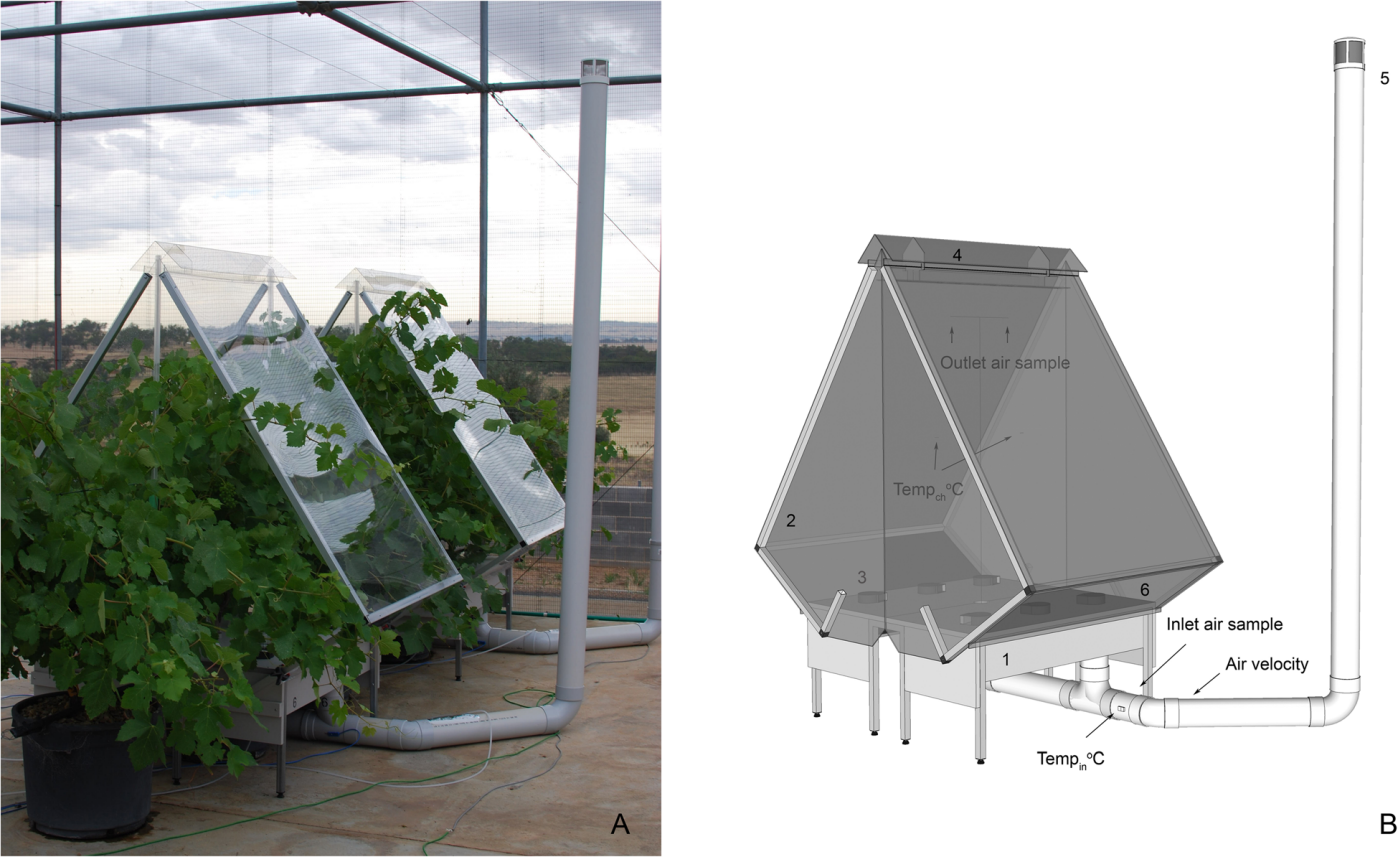
Ambient air supplied gas exchange chambers installed on potted Shiraz grapevines (**a**). Design details (**b**) showing the two-part chamber base (1), two part transparent acrylic chamber (2), six internal blower fans (3), air exhaust cover and baffle (4), ambient air intake (5) and removable lower side panels for fruit sampling (6). Additional text indicates position air velocity sensor, air sampling and temperature sensor on the inlet side, and two points of internal air sampling and air temperature measurement

For whole canopy gas exchange chambers previously used with grapevine studies [20–27], designs have ranged from simpler ‘balloon’ type designs that enclose the entire canopy with a transparent plastic film [24, 25], to fully framed chambers that provide greater resistance to wind [26, 27]. The design of the chambers in the present study was primarily influenced by the canopy shape, which was wide relative to the length of the cordon, and therefore most effectively enclosed by a rectangular shape (Fig. 1a). Fabrication of the chamber tops from solid plastic panel avoided issues with damage from wind and trimmed shoots on the outside edge of the canopy. From observations during smoke tests, air mixing and movement through the chambers was uniform despite the rectangular shape, and the horizontal air entry from the six internal fans mixed the air underneath the vine before rising vertically through the canopy. The time taken for smoke to clear the chamber was approximately 1 min with the 2.6 m^3^ min^− 1^ daytime air flow rate, and the air flow did not result in any obvious leaf movement except where lower shoots hung in the immediate vicinity of the fan outlets. Although no attempt was made to describe the effects of solar elevation and reflection from the panels in detail, a comparison between photosynthetic photon flux density (PPFD) measured above the wire of the bird-proof enclosure with a sensor placed underneath a horizontal sheet of the same acrylic plastic showed just over 80% transmission at solar noon. With PPFD values of 2100 μmol m^− 2^ s^− 1^ usually attained on a clear summer day, light conditions within the chambers would be at, or close to light saturated.

Air flow rates through open gas exchange chambers represent a compromise between minimizing internal temperature rise above ambient and maintaining sufficient CO_2_ differential for accurate photosynthesis measurements. In a previous study with field-grown grapevines it was estimated that the internal temperature of a sun exposed 8 m^3^ chamber could be expected to rise + 3 °C above ambient with two full air volume exchanges per minute [26]. Measured values with chambers designed to this specification were found to be consistent with this estimate, with temperatures at canopy height inside the chamber within 2.5 °C of the same position outside the chambers. Similarly, in another study with field grown grapevine, Pagay [23] reported a maximum rise of + 3 °C under clear conditions for 2.5 m^3^ volume chambers. For chambers built for large pot-grown vines, Poni et al. [21] reported an average temperature rise for 0.6 m^3^ volume chambers of + 1.8 °C and + 2.4 °C for well-watered and water stress treatments, respectively. The authors of the latter study also suggested an alternative design guideline of a minimum of approximately 3 to 4 L s^− 1^ of air flow per square meter of leaf area to avoid overheating.

**Fig. 2:**
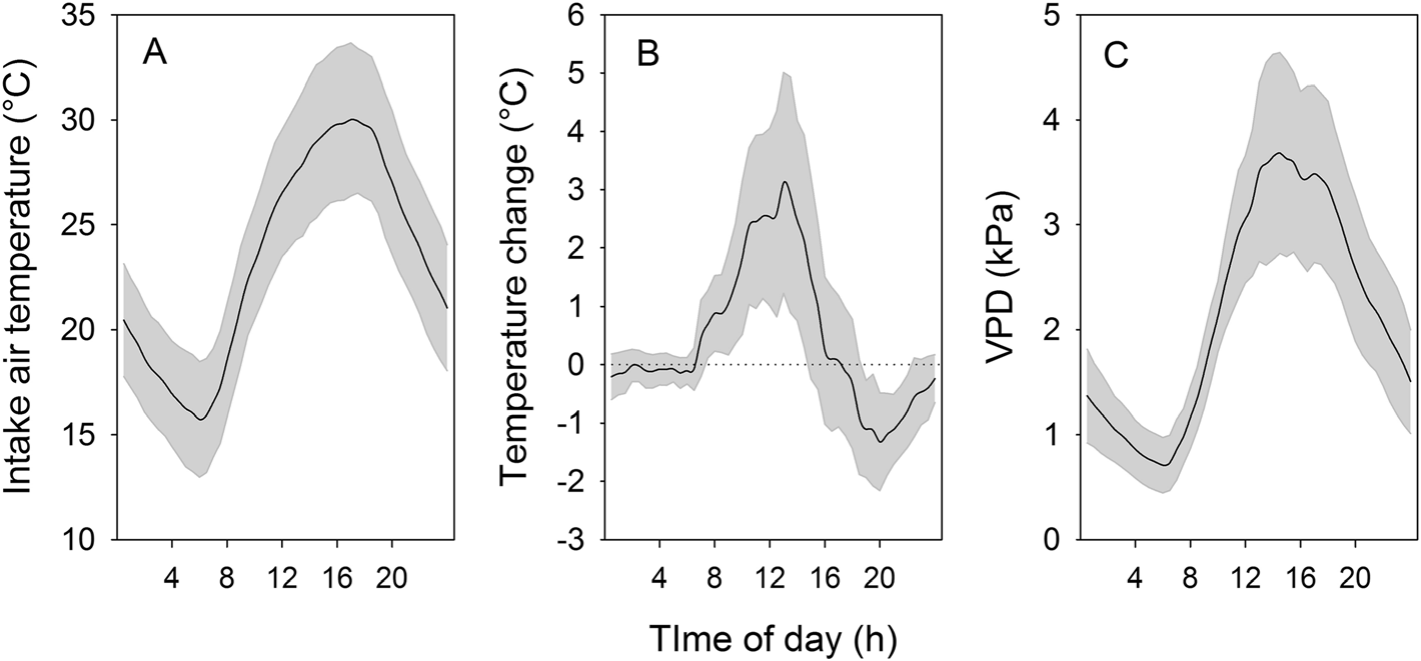
Diurnal pattern of ambient intake air temperature (**a**), temperature change from inlet to outlet (**b**), and vapour pressure deficit (**c**) of supply air for the three ambient air supplied chambers from December 262,013 to January 82,014. Shaded area ± standard deviation

For the three chambers configured for ambient air supply in the present study, with 2.2 air volume changes per minute, the mean maximum temperature rise over 14 days was + 3.5 °C above the inlet temperature, and mean daily temperature rise + 1.4 °C (Fig. 2). With an average leaf area of 4.49 m^2^ in the ambient treatment (Table 1), the air flow rate relative to leaf area was 9.7 L m^− 2^ s^− 1^ and more than double that suggested [21]. However, given the otherwise similar temperature rise within the chambers to previously described systems, higher air flow rates appeared to be required for the location of the study which is characterized by high summer temperatures and solar radiation. The chamber temperature increase closely followed light intensity, reaching a peak at solar noon and then cooling below the inlet air temperature as the PPFD fell below 1000 μmol m^− 2^ s^− 1^ in the late afternoon. While the maximum temperature rise was higher than reported for other chambers systems, the mean inlet chamber temperature was 1.3 °C cooler than the air temperature measured at approximately the same height outside the chambers. The mean internal chamber temperature of 24.0 °C across the 14 days was therefore close to the mean ambient temperature of 23.8 °C. This may reflect the greater height of the intake chimneys above the concrete base of the bird-proof enclosure, which at 1.8 m above the ambient temperature sensor, was in more open conditions above the canopy.

**Fig. 3:**
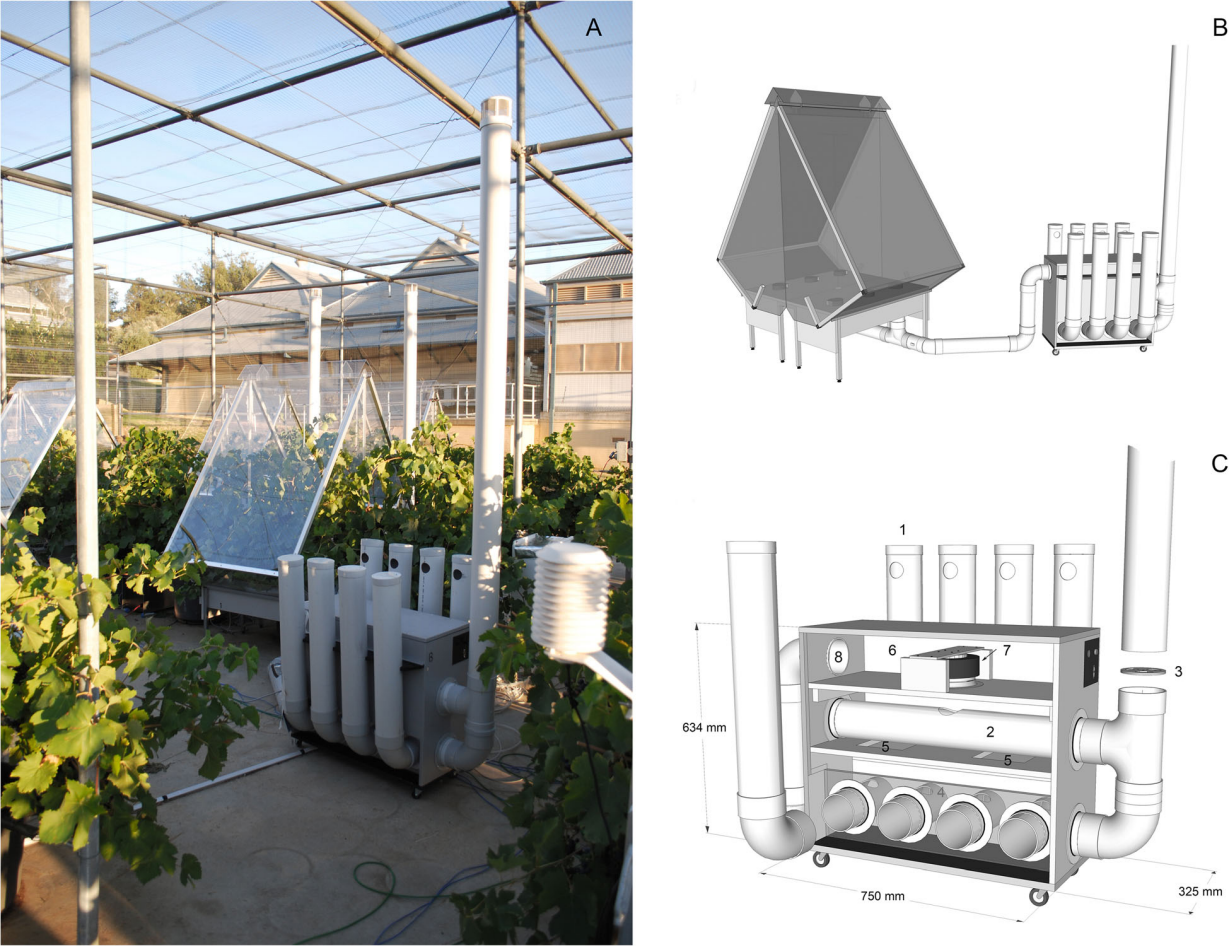
Gas exchange chamber with CO_2_ scrubber connected (**a**, **b**). Design details (**c**), indicate intake for soda lime scrubbing tubes (1), ambient by-pass air and manual control valve (2,3), lower chamber of fully scrubbed air (4), open-cell foam air filters (5), mixing chamber for by-pass and scrubbed air (6), centrifugal fan (7) and outlet to chamber (8). The lower pipe fitting below the by-pass valve is only included for support of the intake chimney, and is not continuous with the lower chamber of the scrubber

**Table 1:** Summary of reproductive and vegetative growth characteristics of three grapevines in each CO_2_ supply treatment

	Harvest^a^ yield	Leaf area^b^	Leaf area to fruit ratio	Pruning weight	Pruning weight ratio
	(kg vine^−1^)	(m^2^ vine^− 1^)	(cm^2^ g^− 1^)	(kg vine^− 1^)	
Ambient	3.76	4.49	11.5	0.66	6.0
Sub-ambient	3.70	4.26	9.8	0.61	6.2
	ns	ns	ns	ns	ns

^a^February 2 ^b^∑Leaf area (cm^2^) = 2.434 × L + 0.855 × L2, where L = leaf length (cm) measured January 9

### CO_2_ scrubber performance and operation

**Fig. 4:**
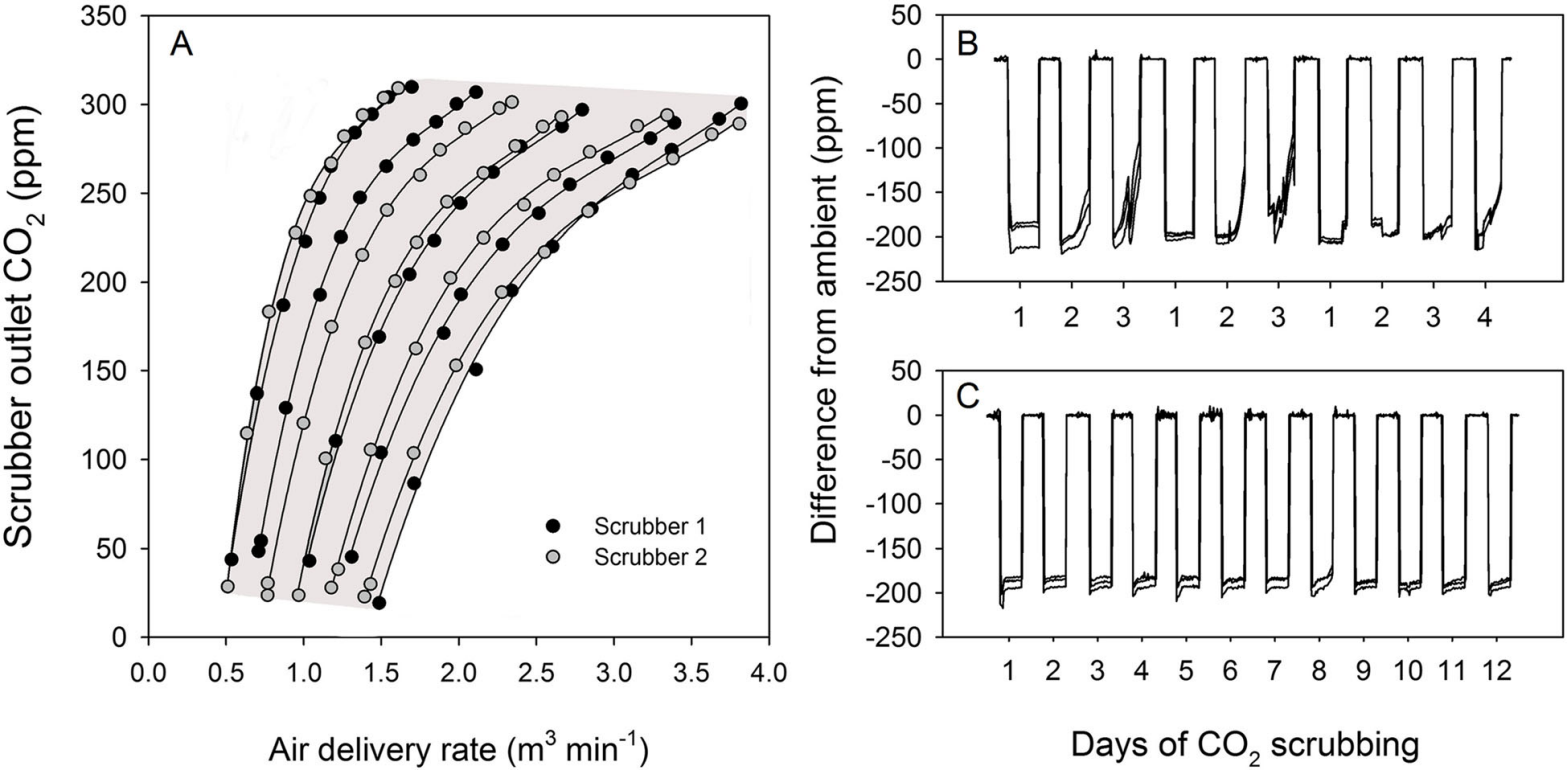
Operating range (grey shaded area) of CO_2_ scrubbers as determined by fan speed and ambient air mixing (**a**). Each fitted line indicates pre-programmed fan speed, and the points down each curve the progressive closure of the ambient air by-pass valve. Concentration difference of CO_2_ from ambient for the 10 day scrubbing period of the veraison experiment described in this paper (**b**), and an example from a subsequent experiment in 2015 (not presented) where the scrubbing tubes were re-wet on a daily basis to replace water lost due to evaporation (**c**)

With fresh soda lime, and all connections fully sealed, the scrubbers had the capacity to deliver air that had CO_2_ completely removed. However, to facilitate refiling the tubes after the soda lime was exhausted, the pipe fittings that were used to construct the scrubbing beds were left as a push-on fit only. When connected to a chamber for normal operation (Fig. 3a), the lowest CO_2_ concentration obtainable was therefore ~ 15 ppm (Fig. 4a). With all flow being directed through the highest resistance pathway of the scrubbing beds, the scrubbers could deliver air with this concentration of CO_2_ at a volume of 1.5 m^3^ min^− 1^. At the other end of the operating range, when unrestricted flow was allowed through the inlet chimney and lowest resistance pathway, the scrubbers could deliver ~ 300 ppm CO_2_ at 3.5 m^3^ min^− 1^. If intermediate values between 300 ppm and ambient were required, then the scrubber tube inlets could be progressively covered to prevent residual air flow through the soda lime. To achieve the targeted 200 ppm reduction from ambient air required for the present study, and to match the intake of the chamber fans such that air flow was not modified relative to the ambient chambers, the scrubbers were run at approximately 50% bypass and close to full fan speed. Previously, Lloyd et al. [19] provided a brief description of a scrubbing system that used 80 kg of soda lime to scrub air at a rate of 12 m^3^ min^− 1^ for large whole tree chambers. This equates to 6.6 kg of soda lime per 1 m^3^ of air, which is comparable to the scrubbers in the present study with a ratio of 5.5 kg of soda lime per 1 m^3^ of air when running at 50% bypass. The six fans within each chamber did have the capacity to pull air through the soda lime beds without the assistance of the scrubber fan if the duty cycle of their control signal was increased. However, the approach used here avoided a large vacuum developing between the chamber and scrubber, and allowed adjustments to be made with the manual scrubber controls rather than changing the microcontroller program that ran the chamber fans.

With the first extended period of use of the scrubbers, a stable 200 ppm reduction in CO_2_ from ambient air could be maintained for 1 to 2 days depending on weather conditions. Forcing a higher proportion of air through the scrubbing beds could prolong this period to some extent, but after 3 or 4 days the soda lime needed replacement (Fig. 4b). Thus, the target reduction of 200 ppm could be achieved with fresh soda lime, but the average treatment difference was reduced to 177 ppm (Table 2). It was subsequently recognized that this loss of scrubbing effectiveness was due to drying of the soda lime rather than the chemical exhaustion of the CO_2_ absorption reactants. For the soda lime reaction, water is required for the first stage when CO_2_ from the air dissolves to form carbonic acid. Subsequent reactions with calcium hydroxide catalysed by sodium or potassium hydroxide lead to the production of calcium carbonate. In the presence of adequate water, the CO_2_ absorption capacity of the soda lime is therefore determined by the utilization of the calcium hydroxide. The Sofnolime® used in the present study had a moisture content of 16–20% according to the manufacturer’s specifications, meaning that each scrubber tube would contain an average of 608 mL of water if filled with fresh absorbent. During the current study, the average water loss from each tube over the 10 day period was 278 mL based on the difference in water vapour concentration between the scrubber air outlet and the intake of the ambient chambers. In a later experiment with the system, where a daily addition of approximately 250 mL of water was made to each tube, a period of 12 days of effective scrubbing was maintained without the need for new soda lime (Fig. 4c). This period ended with the completion of the sub-ambient treatment rather than exhaustion of the soda lime.

**Table 2:** Summary of sub-ambient CO_2_ effects on daily canopy gas exchange parameters and water use efficiency (WUE) from December 26 to January 8

		CO_2_ exchange (g vine^−1^ day^−1^)			Water use (L vine^− 1^ day^− 1^)			WUE (g C0_2_ L^− 1^ H_2_O)
	Average [CO_2_]	Day	Night	Net	Day	Night	Total	
10 day treatment period								
Ambient	412 ppm	69.0	−3.8	65.2	9.0	0.53	9.5	7.1
Sub-ambient	235 ppm	33.2	−3.1	30.0	10.1	0.48	10.5	3.0
Change		−52%	− 20%	−54%	+ 12%	− 11%	+ 11%	−58%
4 day treatment period								
Ambient	408 ppm	77.4	−3.0	74.4	9.0	0.50	9.5	7.9
Sub-ambient	408 ppm	76.6	−2.6	74.0	8.9	0.55	9.5	7.9
10 days	Treatment	0.003	0.100	0.005	ns	0.086	ns	0.002
	Day	< 0.001	0.012	< 0.001	< 0.001	< 0.001	< 0.001	< 0.001
	Treatment x Day	0.008	ns	0.007	ns	0.023	ns	< 0.001
4 days	Treatment	Ns	ns	ns	ns	ns	ns	ns

**Fig. 5:**
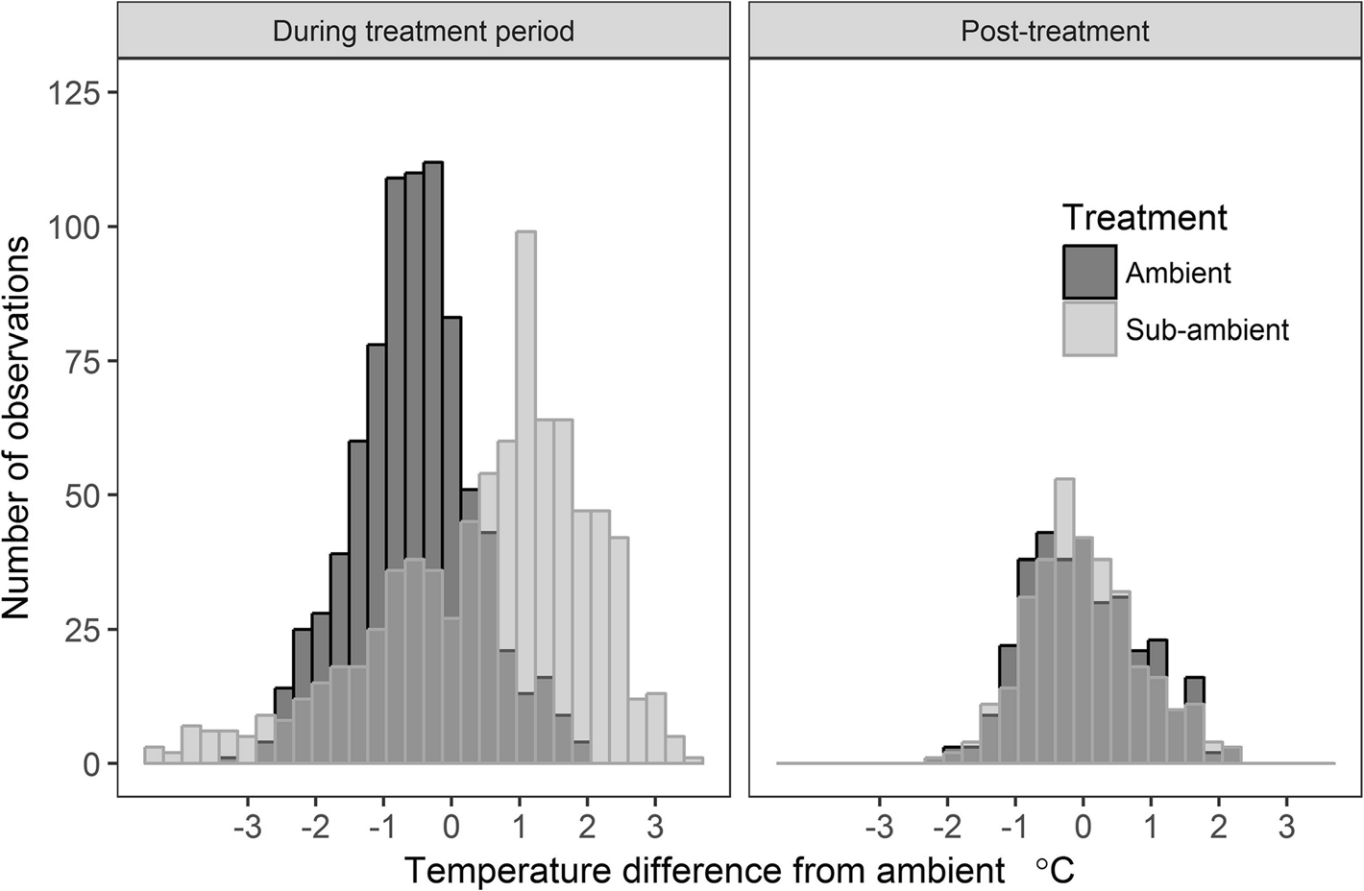
Difference in average daytime chamber air temperature from ambient during the 10 day period of CO2 scrubbing and 4 day post-scrubbing period

In addition to a gain in water vapour, the temperature of supply air also increased in transit through the CO_2_ scrubbers. While not specifically apportioned to either effect, this was likely due to a combination of solar heating on the scrubber itself and the exothermic soda lime reaction. To compare the possible implications of this temperature rise for the fruit microclimate, an average of the inlet and outlet temperature has been used to approximate the temperature at the fruiting-zone at approximately halfway between the fan outlets and the measurement point trellis foliage wire. For the 10 day scrubbing period, the mean day time temperature for the fruiting zone was 27.2 °C for the ambient chambers and 28.2 °C for the sub-ambient chambers, with a difference between treatments of 1.04 °C (Fig. 5). Including overnight temperatures, where the scrubbers were disconnected from the chambers, the mean overall temperature difference between treatments was 0.68 °C.

### Whole canopy photosynthesis and transpiration

**Fig. 6:**
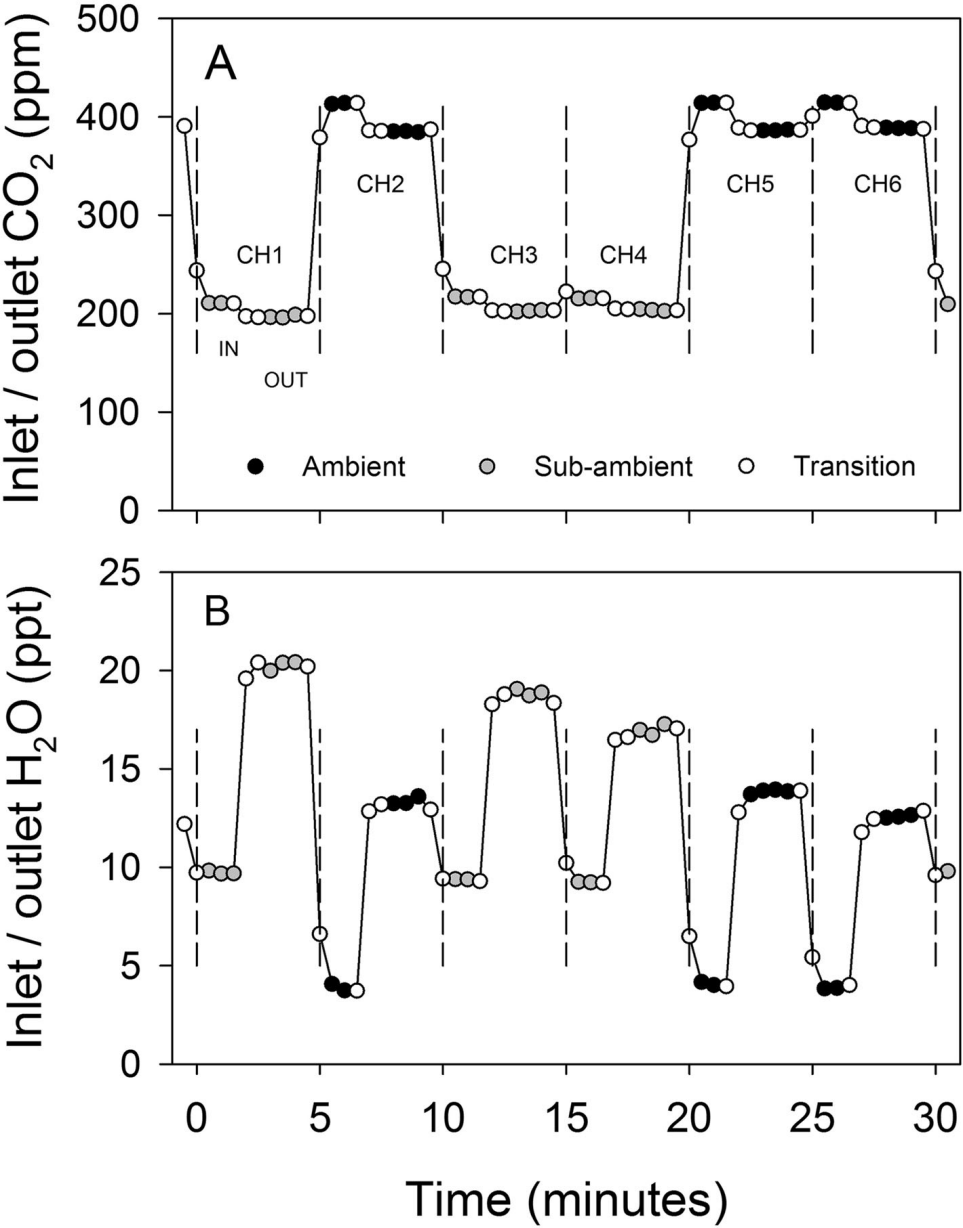
Example 30 min measurement cycle of the six chamber system showing inlet and outlet CO_2_ concentration (**a**) and corresponding water vapour concentrations (**b**). Filled symbols indicates values used for subsequent gas exchange calculations

During the 10 day scrubbing period, the mean CO_2_ depletion on transit through the chambers for one hour either side of solar noon was 23.6 and 12.1 ppm respectively for the ambient and sub-ambient treatments. An example period of gas exchange measurements for a full cycle of 6 chambers in shown in Fig. 6. For the ambient chambers, individual CO_2_ concentration differences between the intake and outlet reached 32 ppm, and for the sub-ambient chambers, 16 ppm. Under ambient CO_2_ supply, the maximum daily average photosynthesis rates were mostly between 9 and 11 μmol m^− 2^ s^− 1^ over the 14 day period with occasional individual values above 12 μmol m^− 2^ s^− 1^. These rates are consistent with those reported for field-grown Cabernet Sauvignon with a similar training system to the one used in the present study [20] and intermediate to values of 2 to 6 μmol m^− 2^ s^− 1^ for shaded leaves and 12 to 17 μmol m^− 2^ s^− 1^ for sun-exposed leaves reported for field-grown vines under comparable irrigated hot climate growing conditions [28, 29]. During the 10 day period of sub-ambient CO_2_, which included two heavily overcast days, the average maximum photosynthesis rates were 6.5 and 11.4 μmol m^− 2^ s^− 1^ respectively for the sub-ambient and ambient treatments. For the whole canopy, these values were 29 and 54 μmol vine^− 1^ s^− 1^, respectively. For other studies with grapevine that have reported assimilation rates on a canopy basis, values of up to 60.7 μmol vine^− 1^ s^− 1^ for field grown Sauvignon Blanc were recorded by Petrie et al. [27], and ~ 30 μmol vine^− 1^ s^− 1^ for pot-grown Sangiovese Poni et al. [21]. With an average leaf area of 4.5 m^2^ for the vines in the present study, and ~ 4.6 and 2.87 m^2^ respectively for these earlier studies, the CO_2_ assimilation rates would also be comparable on a leaf area basis.

**Fig. 7:**
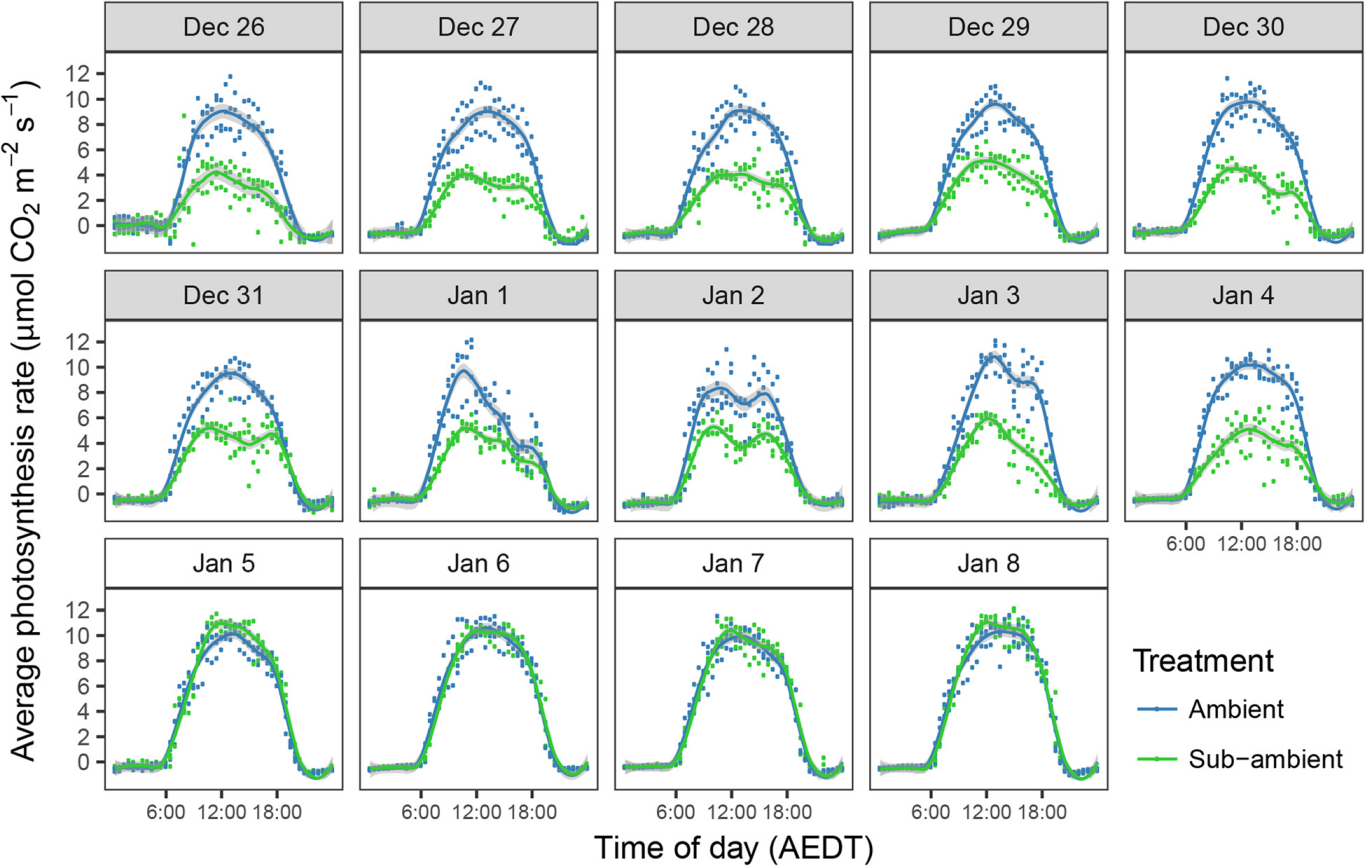
Average diurnal canopy photosynthesis rates for ambient sub-ambient CO_2_ supplied chambers (*n* = 3) for 10 days from December 26, 2013, and 4 days following the return of the sub-ambient chambers to ambient air supply on January 5. Measurement points shown in relation to Australian Eastern Daylight Time (AEDT). The corresponding supply CO_2_ difference for sub-ambient to ambient in shown in Fig. 4b

**Fig. 8:**
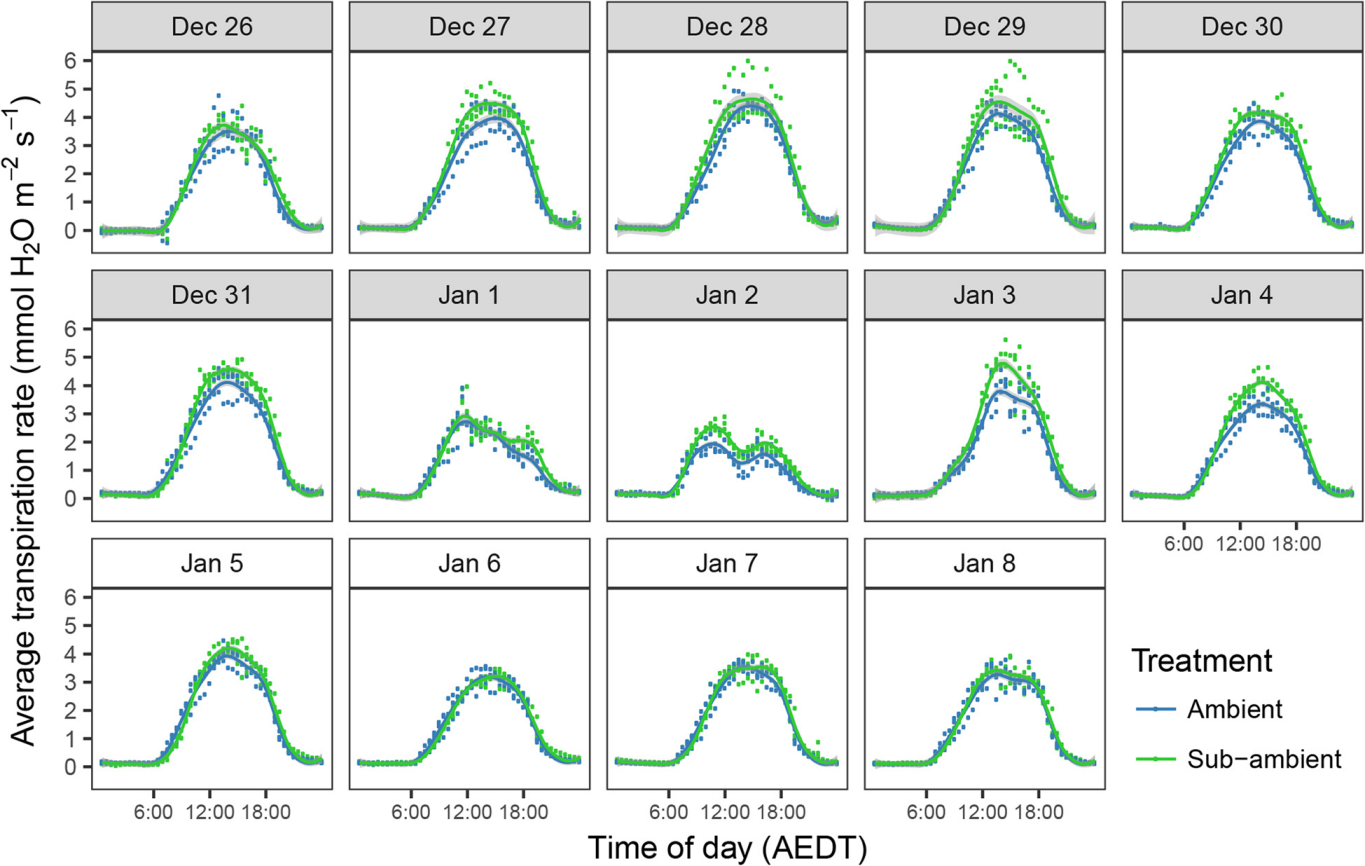
Average diurnal canopy transpiration rates for ambient sub-ambient CO_2_ supplied chambers (*n* = 3) for 10 days from December 26, 2013, and 4 days following the return of the sub-ambient chambers to ambient air supply on January 5

The daily time course of CO_2_ and H_2_O fluxes are shown in Figs. 7 and 8, respectively. The corresponding average daily sums for CO_2_ exchange and water use are shown in Table 2. The average net daily CO_2_ assimilation over the 10 days was 65.2 g for the ambient vines and 30 g for the sub-ambient, representing a reduction in carbon assimilation of 54%. By slowing the airflow through the chambers at night, the system was also able to resolve the 1 to 3 ppm rise in CO_2_ due to respiration. These rates were higher early in the night than pre-dawn, and although not clearly visible at the scale shown in Fig. 6. With mean night temperature used a covariate, which was significant at *P* = 0.029, there was also a weak significant difference in total respiration between treatments with vines in the sub-ambient chambers losing an average 3.1 g of CO_2_ compare to 3.8 g in the ambient chambers. At 10.5 and 6.0% of the day time CO_2_ assimilation, respectively, this had minimal effect on the net daily balance of CO_2_ exchange, but indicates a possible effect of carbohydrate (CHO) availability on night respiration. It has previously been demonstrated that dark respiration is correlated with leaf CHO fractions and in turn photosynthesis in the preceding light period [30]. Under elevated CO_2_, leaf CHO and dark respiration can increase in some species [31] suggesting that reduced dark respiration of vines following the day period at sub-ambient CO_2_ in the present study could be due to a lower concentration of leaf CHO at the end of the day.

Under elevated CO_2_, stomatal conductance has in most cases been observed to decrease, leading to reduced water use on both a leaf and whole canopy basis [31, 32]. Reduced stomatal conductance has also been observed with mature field-grown grapevines under elevated CO_2_ [29, 33]. Conversely, if CO_2_ concentrations are lowered, stomatal conductance would be normally expected to increase [34, 35]. Although stomatal conductance was not measured in the current study, there was a trend for increased canopy water use across the 10 days of sub-ambient CO_2_ treatment which would suggest an underlying stomatal response (Table 2). As the air supply from the scrubber was slightly warmer with a day time average of 28.2 compared to 26.8 °C for the ambient supply chambers for the first 10 days shown in Fig. 6, it is possible that the transpiration increase could partly reflect the differences in chamber environmental conditions. However, with the evaporation of water from the soda lime beds, supply air water vapour concentration co-varied with temperature. The mean increase in vapour pressure deficit (VPD) was therefore offset to some extent, with average day time values of 2.87 and 2.68 kPa for the sub-ambient and ambient chambers respectively.

The identical daily water use for both treatments as the sub-ambient chambers returned to ambient air supply in the last 4 days of the experiment would indicate that the 11% increase in transpiration during the scrubbing period was real. For future studies where stomatal responses and transpiration were to be the main focus, CO_2_ could be scrubbed down to the sub-ambient target across all chambers and then re-injected to avoid the small temperature differences associated with the CO_2_ scrubbers. There may be some remaining effect of stomatal responses to CO_2_ on transpiration and VPD, which can be managed in chambers with re-circulated air [36], but would need further consideration with the open design used here. Irrespective of cause and significance, the slight rise in water use, coupled with the large decrease in photosynthesis under sub-ambient CO_2_, meant that water use efficiency was more than halved over the 10 day period from an average of 7.1 to 3.0 g of CO_2_ assimilated per litre of water transpired (Table 2). As with all other parameters there was no significant difference between treatments on return of all chambers to ambient CO_2_ supply, which would indicate that the results here reflect the gas exchange responses of the initial canopy, and the 10 days was not sufficient for acclimation or shoot growth responses when the canopy was already fully established.

### Berry sugar accumulation

At the onset of ripening, unloading of sucrose from the phloem in grape berries transition from symplastic to apoplastic [37]. Following import from the phloem, this sucrose is split into fructose and glucose by cell wall or vacuolar invertases and then stored in the vacuole. The combined total concentration of these sugars typically reaches 200 to 300 g L^− 1^ at maturity [38]. In the juice collected from the berry samples, fructose and glucose concentrations were significantly lowered by 10 days of sub-ambient CO_2_ supply (Table 3), indicating a reduction in sucrose translocation to berries as a result of reduced photosynthesis. However, despite the 54% reduction in net canopy photosynthesis, the sub-ambient treatment only reduced sugar concentration by 36%, with a gain of 30.8 g L^− 1^ over the 10 days compared to 48.5 g L^− 1^ under ambient CO_2_. On a whole vine basis the sugar accumulated by the fruit in the ambient treatment was 223 g, representing just over 50% of assimilated C during the period. In contrast, fruit in the sub-ambient treatment accumulated 166 g of sugars or an amount equivalent to 72% of the C assimilated by photosynthesis during the period.

**Table 3:** Summary of berry composition changes following 10 days of sub-ambient CO_2_ supply, and comparison of total canopy carbon assimilation with berry sugar accumulation during the corresponding period

	Berry composition comparison						Source-sink parameters		
	Soluble solids (°brix)		Glucose (g /L)		Fructose (g/L)		Carbon assimilation (g vine^−1^)	Berry sugar gain	Berry C / assimilated C
	Dec 26	Jan 5	Dec 26	Jan 5	Dec 26	Jan 5	CO_2_	(g vine^−1^)	%
Ambient	5.6	11.5	14.6	51.3	6.9	43.7	652	223	50
Sub-ambient	5.4	9.8	16.7	42.1	8.3	35.2	302	166	72
Treatment	ns	**	ns	**	ns	**	***	ns	*

Although not assessed in the current study, carbohydrate reserves, and particularly those of the root system, can provide an alternative C supply for ripening fruit [39, 40]. Under heavy fruit load, the growth of new fine roots and C allocation to the roots can also be reduced [41]. Thus, the mobilization of stored C, or diversion of a greater percentage of current assimilate away from vegetative parts of the vines, may have been sufficient to offset some of the effects of reduced photosynthesis on berry sugar accumulation. While upregulation of photosynthesis was a possibility based on findings of earlier studies [eg. 15] net canopy C assimilation was measured here and shown to be reduced by over 50%. The relative increase in the proportion of C accumulated by the fruit therefore highlight the high sink strength of developing berries at véraison, and their capacity to compete for C under source limitation.

## Conclusion

The combination of custom built soda lime-filled CO_2_ scrubbers and whole canopy gas exchange chambers was found to be an effective method for reducing the net carbon assimilation of grapevine canopies. Even at the early stage of berry ripening, which was just commencing when the chambers were installed, the reduction in canopy photosynthesis resulted in a significant reduction in berry sugar accumulation. At the concentrations used in the present study, where CO_2_ was reduced to an average of 235 ppm compared to ambient at 412 ppm, there was an average 52% reduction in day time carbon assimilation which was consistent with the near linear rubisco response to CO_2_ across this range. While the CO_2_ scrubbers developed in this study were designed for the purpose of slowing berry sugar accumulation and could maintain sufficient air flow across a range of approximately 180 to 300 ppm, any desired range could be achieved with a combination of two scrubbers connected in parallel and additional CO_2_ injected back into the air supply. This would extend the potential application of the system to increasing as well as decreasing photosynthetic carbon supply, and extend the scope to source and well as sink-limited studies.

## Materials and methods

### Location and grapevine growing conditions

All work described in paper was undertaken at the National Wine and Grape Industry Centre, Charles Sturt University in Wagga Wagga, New South Wales, Australia (35.06 °S, 147.36 °E), and utilized an existing population of large potted Shiraz grapevines (*Vitis vinifera* L., clone PT23) that were located in a bird-proof wire mesh enclosure. The vines were planted on own roots in 2008, and trained to single spur-pruned bilateral cordon on a fixed fruiting wire 80 cm from the ground. A second fixed wire at 120 cm above the ground provided support for the canopy. The pots were 52 L in volume (500 mm wide × 380 mm high), and arranged with 1 m spacing in rows of 5 vines in an E-W alignment with 3 m between rows. Pots were filled with a commercial bulk-composted potting mix, and fertilized manually with diluted liquid fertilizer (Megamix Plus®, RUTEC, Tamworth) and additional applications of magnesium sulphate and gypsum. Irrigation was provided via a 4 L h^− 1^ pressure compensated emitter installed on each side of the trunk to provide water to two sides of the root system. Irrigation was controlled by an automated timer, with the schedule and duration of three to four daily irrigations adjusted manually according to seasonal water requirements. Shoot numbers ranged from approximately 30 to 38 per vine, providing a sprawl canopy with similar structure and density to field-grown grapevines.

### Gas exchange chambers

Six gas exchange chambers were constructed with transparent acrylic tops that incorporated a lightweight plastic and aluminum frame (Cubelok, Capral Limited, Parramatta, Australia), and a base made from polyethylene plastic panels and a galvanized steel frame. The chamber top and base were both designed in two halves that could be installed from each side of the trellis with minimal impact on the shape of the canopy (Fig. 1). The chamber top enclosed a volume of 1.2 m^3^ and was constructed from flat sheets of 3 mm thick acrylic on the sides parallel to the vine row, and 4 mm thick on the two sides perpendicular to the vine row. Under clear sky light transmission between 400 and 700 nm was 95%. An 85 mm gap between the top of the 3 mm sheets allowed outgoing air to exit the chamber, with an overlapping ridge cap supported 24 mm above the chamber sides to minimize ambient air intrusion during windy conditions. When installed, the maximum outside dimensions of the assembled chambers were 1620 mm perpendicular to the row, 1000 mm parallel to the row and 2000 mm high.

The external panels of the chamber base were constructed from 19 mm plastic boards made from a combination of high density polyethylene and polypropylene (UniboardECO, Dotmar Plastic Solutions, Sydney, Australia). On the top of each half of the chamber base, three 24 VDC blower fans (San Ace B97, Model 9BMB24P2G01, Sanyo Denki Co. Ltd., Tokyo, Japan) were mounted against a 64 mm diameter hole, and angled to mix and distribute air in a horizontal plane across the base of the chamber. An air-tight rectangular box made from a combination of 12 mm and 6 mm sheets of the same plastic was fixed to the underside of each chamber base half to enclose the air intake of the three fans. A port on the underside of each of the boxes was then connected to a common air intake made of 105 mm internal diameter (nominal size 100 mm) polyvinyl chloride (PVC) pipe and compatible fittings. This air intake could either be attached to a 3 m high chimney for ambient air, or to the outlet of the CO_2_ scrubber described in the next section. The air inside the chamber was then displaced vertically through the canopy and exited through the vent at the top of the chambers. Smoke tests (DATAX, BJÖRNAX AB, Nora, Sweden) were used to assess the uniformity of air flow through the chambers. Removable panels on the lower face of the acrylic chamber tops allowed access to fruit on both side of the vine for berry sampling. Adhesive tape was used to seal the gaps created by the trellis wires between the chamber halves, and adhesive foam weather strips were used between the panels of the chamber top and base.

The speed of the 6 fans was controlled by a pulse width modulation (PWM) signal from an Arduino® UNO compatible micro-controller mounted inside the intake box in one side of the chamber base. During the day, the PWM signal was programmed to a duty cycle of 25%, and an input signal from a manually operated switch was used to slow the PWM to a duty cycle of 10% at night. For the day speed, this equated to a flow rate of approximately 2.6 m^3^ per minute, or 2.2 chamber volumes per minute, and at night, 1.6 m^3^ per minute or 1.3 chamber volumes per minute.

### CO_2_ scrubbers

A portable CO_2_ scrubber was designed so that it could be attached to a chamber, in place of the ambient air intake chimney and reduce the CO_2_ concentration of supply air by approximately 200 ppm compared to ambient air (Fig. 2). Three scrubbers were constructed from a combination of 15 mm plywood and 100 mm nominal diameter (105 mm ID) PVC pipe and fittings that completely scrubbed all CO_2_ from incoming air and then mixed this back in with ambient air to obtain the desired CO_2_ concentration. Each scrubber contained a total of 27 kg of soda-lime (Sofnolime®, Molecular Products Limited, Harlow, UK), divided equally across eight tubes of 100 mm diameter and 480 mm depth that were connected in parallel. Air was pulled through the soda-lime beds, and then an open-cell foam filter under vacuum using a single 24 VDC centrifugal fan (SanAce C175, Model 9TG24P0G01, Sanyo Denki Co. Ltd). A manually operated valve was used to allow some air from an ambient inlet chimney at 3 m to by-pass the scrubbing beds on the upstream side of the fan. Fan speed was controlled by a PWM signal in the same manner as those in the chamber bases, but a potentiometer was used instead of a switch to provide variable control over the fan speed. Through a combination of ambient air bypass and fan speed control, the design allowed variable control over the airflow rate and CO_2_ concentration range. To minimize temperature differences between the ambient and low CO_2_ treatments, scrubbers were covered with reflective foil to reduce solar heating, and ice placed on top of scrubbers and replaced during the day as required.

To test the full operating range of the scrubbers, a series of measurements were made between the minimum and maximum fan speeds with the bypass valve closed progressively until the maximum amount of air possible was forced through the scrubbing beds. A portable infrared gas analyser (Li400XT, LiCOR Biosciences, Lincoln, Nebraska, USA) was used to measure the concentration of outgoing CO_2_, and the process was repeated with a second scrubber.

### Gas exchange measurements

When assembled and running as a complete system with six chambers, the CO_2_ and H_2_O concentrations of incoming and outgoing air were recorded on a 30 min cycle, providing 48 measurement points in a 24 h period. During the measurement period for each chamber, air was sampled simultaneously from the chamber inlet and outlet at a rate of approximately 2 L min^− 1^ via 10 m of 6 mm external diameter polyethylene tubing using a pair of diaphragm pumps (SP550 EC-BLa, Schwarzer Precision GmbH + Co. KG, Essen, Germany) connected in parallel on each line. As shown in Fig. 1b, the inlet air sample was drawn from the PVC air intake tube on the downstream side of the intake fans. The outlet sample was taken from two points within the chamber at 20 cm below the air outlet and 30 cm apart to provide an average air sample. For both inlet and outlet lines, the two pumps were connected in parallel across the outlets of six individually switchable solenoid valves (V2 miniature pneumatic solenoid valve, Parker Hannifin Corp., Cleveland, Ohio, USA) to avoid dead-air volumes and maximize flushing between cycles. These pumps then delivered the air to a 50 mL buffer container that was then sub-sampled via a switchable solenoid valve by a single pump (SP550 EC-BLa) at a rate of approximately 800 mL min^− 1^ for subsequent CO_2_ and H_2_O measurements. Excess air from the 50 mL buffer volumes vented to atmosphere.

Carbon dioxide and H_2_O vapour concentrations were measured at 5 s intervals using an infrared gas analyser (Li840A, LiCOR Biosciences, Lincoln, Nebraska, USA) set to 50 °C and the average value recorded at 30 s intervals with an external data logger (CR1000, Campbell Scientific, Logan, Utah, USA). For the inlet air, these measurements were made for 2 min, and for the outlet air sample, 3 min. A relay controller (SDM-CD16AC, Campbell Scientific) was used to drive the progressive switching of the six pairs of solenoid valves at 5 min intervals, and within that period, to switch the single sub-sampling solenoid from the chamber inlet to outlet after 2 min. Data points recorded during transition periods were removed during subsequent processing, leaving two inlet measurements and four outlet measurements to average for subsequent gas exchange calculations.

Chamber and external environmental parameters were recorded with a second data logger and multiplexer (CR1000 and AM2 5 T, Campbell Scientific, Logan, Utah). Air inlet temperature was measured with T-type thermocouples (24 AWG) at a single point inside the inlet tube under the chamber base, and at two outlet points on each side of the canopy (wired in parallel for average value) from under white plastic radiation shields attached to the fixed foliage wire. Air velocity was recorded in the centre of the inlet tube with a hot-film element sensor (EE576 or EE671, E + E Elektronik, Engerwitzdorf, Austria). Photosynthetically active radiation outside the chamber was recorded with Quantum sensor (LI-190R, LI-COR Biosciences), and temperature and relative humidity (HMP-50, Vaisala, Helsinki, Finland) inside the bird-proof enclosure were recorded at the same intervals as the chamber measurements.

### Sub-ambient CO_2_ experiment

At the first visual indication of berry colour change in late December 2013 the chambers were installed on the canopies of six grapevines selected from the larger population in the bird-proof enclosure. For a period of 10 days from December 26 three chambers were supplied with ambient air, and three chambers connected to the CO_2_ scrubbers and supplied with air at a target of 200 ppm below ambient during daylight hours. While no pre-treatment comparison was able to be made due to an earlier expected onset of véraison, from January 5 all chambers were run at ambient CO_2_ for an additional 4 days to compare them in the post-treatment period. When the chambers were removed, the length of every leaf from each vine was recorded. Using a regression established between leaf area, measured (LI-3100C, LI-COR Biosciences), and length, with leaves sampled destructively from vines that had not been used in the chambers, the total leaf area of each vine was then calculated.

### Berry sampling and analysis

Berry samples collected on the first day of the 10 day scrubbing period, and then on the morning of the eleventh day, were used to assess the effect of the sub-ambient treatment on berry sugar accumulation. Accessed via removable panels on the side of the chambers, 20 random berries were collected from each side of the vine to provide a 40 berry sample. The samples were immediately weighed, separated into skin and seeds over ice and then frozen in liquid nitrogen. The pulp was manually homogenised while cooled over ice, and juice separated and 1 mL sub-samples frozen in liquid nitrogen. Remaining juice was used to determine soluble solids. All samples were stored at − 80 °C prior to subsequent analysis. Juice samples were thawed, vortexed to mix and filtered to 0.22 μm and fructose and glucose concentrations determined by HPLC-RI on a 300 mm × 7.8 mm Aminex HPXM87H ion exclusion column (BioMRad Laboratories, Berkeley, USA) using the method of Frayne [42].

To estimate the total amount of sugar accumulated by each treatment in the 10 days between the first and last berry sampling, sugar content per berry was calculated using the weight to volume relationship for Shiraz described by Gray and Coombe [43]. Fruit from all vines was harvested and weighed on February 12, and using harvest sampling berry weights, the total number of berries per vine was calculated. Allowing for the removal of berries at each sampling date, an estimate of total sugar concentration per vine could be made based on total berry volume and juice sugar concentration.

### Gas exchange calculations

Volumetric air flow was calculated from the inlet air velocity measurements for the 5 min period of gas exchange for each chamber and converted to molar air flow (Eq. 1) as per Long and Hallgren [44], where *u*_*e*_ = molar flow of air (mol s^− 1^), *fv* = volumetric air flow (cm^3^ min^− 1^), 22.4 = volume (dm^3^) of one mole of air at standard temperature and pressure of 273.15 K and 101.3 kPa respectively, T = air temperature (°C), and P = atmospheric pressure (kPa). The inlet thermocouple for each chamber was used for the air temperature, while atmospheric pressure was obtained from the Australian Bureau of Meteorology Wagga Wagga airport weather station (35.16 °S, 147.46 °E), situated approximately 15 km south east of the experiment location.

$$ {u}_e=\frac{f_v}{1000}.\frac{1}{22.4}.\frac{273.15}{\left(273.15+T\right)}.\frac{P}{101.3}.\frac{1}{60}\kern5.25em (1) $$

Average canopy transpiration (E; Eq. 2), photosynthesis (A; Eq. 3) and rates were calculated using the following equations [45], were s = leaf area (m^2^), w_e_ and w_o_ = inlet and outlet vapor concentration (mol mol^− 1^) respectively, *Δ* = difference between inlet and outlet CO_2_ concentrations (mol mol^− 1^) and c_e_ = inlet CO_2_ concentration.

$$ \kern0.75em E=\frac{u_e}{s}.\frac{\left({w}_o-{w}_e\right)}{\left(1-{w}_o\right)}\kern5.5em (2) $$

$$ \kern0.5em A=\frac{u_e}{s}.\frac{\left(1-{w}_e\right)}{\left(1-{w}_o\right)}.\varDelta -E.{C}_e\kern1.5em (3) $$

Outlier values, screened based on the ratio of the inlet and outlet CO_2_ concentrations and to allow daily sums to be calculated, were replaced with the average of two adjacent readings for that chamber. An outlier or potentially incorrect reading was defined as outlet concentration that was more than 10% lower than the inlet during the day, or an outlet concentration more than 2% higher at night. Only 25 points from 4032 measurement points over 14 days required replacement with this method, and in most cases these could be explained by berry sampling, or the connection or disconnection of the CO_2_ scrubbers inadvertently coinciding with the 5 min monitoring period for each chamber.

### Statistical analysis

For the gas exchange data, the three chambers in each treatment were blocked according to the leaf area of each vine and analysed as a split plot experiment with treatment as a main plot and time as the subplot factor using Genstat v18 (VSNI, Hemel Hempstead, England, UK). Mean chamber temperature inside the chamber was used as a covariate for comparison of night respiration rates. The 10 day period with the ambient and sub-ambient treatments was analysed separately from the 4 day period. Treatment means for single measurements were compared using Student’s T-test.

## Data Availability

The datasets used and/or analysed during the current study, as well design files for the chambers and CO_2_ scrubbers, are available from the corresponding author on reasonable request.

## Abbreviations

CHO: carbohydrate
CO_2_: carbon dioxide
PPFD: photosynthetic photon flux density
PWM: pulse width modulation
VPD: vapour pressure deficit
